# Tailoring cardiac environment in microphysiological systems: an outlook on current and perspective heart-on-chip platforms

**DOI:** 10.4155/fsoa-2017-0024

**Published:** 2017-05-03

**Authors:** Giovanni Stefano Ugolini, Roberta Visone, Daniela Cruz-Moreira, Alberto Redaelli, Marco Rasponi

**Affiliations:** 1 Department of Electronics, Information & Bioengineering, Politecnico di Milano, Milan, Italy

**Keywords:** 3D cell culture, cardiac models, cell microtissues, heart-on-chip, microfluidics, microphysiological systems, organs-on-chip

With cardiovascular diseases representing a leading cause of death worldwide, several biological aspects of heart physiology and pathology remain open and therapeutic needs widely unmet. The attention of biomedical research has been directed toward the development and exploitation of meaningful *in vitro* models of the myocardium, in both health and disease. Indeed, an increasing need has risen for advanced platforms able to investigate heart pathological mechanisms or development of drug treatments with low impact on cardiac function.

An ideal *in vitro* heart model should be capable of tailoring environmental cues provided to human cells or tissues (biochemical signals, physical stimulations) while also providing means to analyze functional readouts (e.g., contractility of the tissue, electrical activity). These specifics are the key to conduct both basic research investigations (e.g., cellular mechanisms of cardiac pathologies) and translational studies (effects of candidate drug compounds on heart functions). A class of culture platforms recently acknowledged as prospective to meet these needs and advance these fields are organs-on-chips.

Exploiting photo and soft-lithography microfabrication techniques, organs-on-chip provide microstructured arrangements of cells recapitulating key organ/tissue functions and supplied by microfluidic channels [1]. The relatively low volume of fluids required to culture cells within these devices makes it possible to focus the use of organs-on-chip toward human cells (primary cells or derived from pluripotent stem cells), thus stepping away from animal sources. These concepts have been applied to heart-derived cells in order to more finely recapitulate the cardiac environment *in vitro* within so-called heart-on-chips. To date, this rapidly growing field accounts for several reports describing heart-on-chip designs [2], which in some instances were validated for carrying out relevant drug screening studies [3]. Heart-on-chip models therefore hold concrete potential for advancing the field of cell culture platform. However, they still require improvements and directions in order to attain meaningful biological models for robustly serving pharmaceutical screenings or basic research. We here comment on existing heart-on-chip microfluidic devices, with an outlook on unmet requirements and future directions for rational technological development.

## Controlling physical & chemical environments

An inherent advantage of microfabricated organs-on-chip is the ability of applying tailored physical and/or biochemical cues to cultured cells, in an attempt to more closely mimic the cardiac cellular microenvironment. A prominent physical stimulus in the heart is the cyclic mechanical loading caused by tissue contractility. The mechanical microenvironment is known to have major effects on cardiac cells and has been shown to drive cellular fate of several heart-related cell types [4].

A substantial number of investigations regarding mechanical loading platforms and biological cellular responses have been carried out by means of microfluidic systems. Flexible substrates allow to culture cellular monolayers and on-chip pneumatic actuation systems typically provide pressure or vacuum-driven substrate stretching. This class of microsystems led to significant developments in the exploration of cardiac cells mechano-responses: embryonic stem cells were shown to exhibit a preferential cardiomyogenic differentiation when subjected to cyclic mechanical stimuli [5]. In the field of fibrotic response, human cardiac fibroblasts were shown to exhibit different responses according to the intensity of mechanical stimuli applied [6,7]. Cardiac myocytes 3D constructs exhibited synchronous contraction only under mechanical stimulation, with unorganized patterns of contraction in the nonstimulated counterparts [8]. Of note, recent technical developments include a design solution for coupling 3D hydrogel-based constructs with cyclic mechanical stimulation in microfluidics [8], thus enabling the fabrication of organs-on-chip that combine the physiological architecture of 3D ECM-based constructs with the possibility of mechanically stimulate them.

Electrical stimulation is another key feature reproduced *in vitro* to better recapitulate the *in vivo* cardiac environment. In the heart, cardiac cells are electrically coupled and continuously subjected to electric currents. Biomimetic electric signals have been demonstrated to enhance conductive and contractile properties of cardiomyocytes and to influence stem cell fate [9]. Several microfluidic platforms have been developed to provide cells with different electrical stimulation patterns, applied alone or in concert with other stimulations (i.e., chemical, mechanical, topological). 2D flat electrodes obtained through electrodeposition [10] or laser ablation [11] technologies can be directly integrated in microfluidic platform surfaces, where cellular monolayers are cultured. Alternatively, 3D electrodes can be formed by injecting conductive compounds in a predesigned compartment of the platform [12] or can be achieved directly inserting physical electrodes (platinum wires, carbon rods, stainless steel) in contact with cell culture medium.

Electric fields applied on cardiomyocytes monolayers successfully provided appropriate environmental cues to maintain cardiac phenotype and contractile function *in vitro*, enhancing cellular elongation, cardiac maturation, beating performances and gap junction organization [11]. In heart-on-chip systems housing 3D cell-laden hydrogels, electrical stimulation allowed, for instance, human pluripotent stem cells [13] derived cardiomyocytes to mature in a tissue-like constructs, enhancing construct organization and conduction velocity.

## Standard systems for cardiac functional readouts

While microenvironmental cues are key factors for aiding cardiac constructs maturation and mimicking physiological or pathological states, another crucial aspect in designing heart-on-chip devices is the inclusion of readout systems for assessing cardiac biological functions. Contractility is the hallmark of heart function and contractile force quantification is one of the most important functional characterizations of cardiac muscle. Over the past decades, enormous efforts have been made to develop techniques that overcome the challenges in assessing the contractile properties of cardiomyocytes. Huebsch *et al.* [14] adopted a force transducer system technique to assess microheart muscle physiology. This technique required harsh manipulations of the tissue: microheart muscles were microdissected, removed from its original platform, hooked and immersed in a bath. Although, often used two-point force assays measure force along a single axis, neglecting multidirectional contraction exerted by cells whose cytoskeleton is unaligned (e.g., immature cardiomyocytes).

Some appealing, noninvasive approaches for force measurement are based on small cantilever deflections. In atomic force microscopy, a small cantilever gently touches a beating cardiomyocyte and its vertical deflection tracks the beating force signal [15]. Nonetheless, atomic force microscopy instrumentation has a low chance of being integrated in compact systems. In a pivotal work, Tanaka *et al.* [16] demonstrated a cheaper solution to measure single-cell contractile force, by using polydimethylsiloxane(PDMS)-based arrays of micropillars that deflect according to the motion of attached cardiomyocytes. Their strategy requires video analysis and mathematical estimations that depend on pillar shape and on cardiomyocytes attachment.

More recently, progress has been made toward high-resolution 2D mapping of cell tractions at cell surface. Traction force microscopy (TFM) has been widely used to characterize single cardiomyocyte contractility [17], exploiting cardiomyocytes ability to wrinkle an elastic substrate during contraction. TFM consists on tracking displacements of nanobeads embedded on a hydrogel substrate. Knowing the mechanical characteristics of the substrate and using mathematical estimations TFM allows to quantify the traction stress exerted by contracting cells. The main advantage of TFM is that it is noninvasive, nonterminal and it only requires basic equipment to assess cardiomyocytes contractile performance. Video microscopy simplifies TFM by removing the need of beads embedded in the substrate: indeed, estimated strain fields of complex cell distortions rely on standalone high-quality image acquisition [18].

Another important challenge related to cardiac model generation has been the investigation of electrophysiological characteristics of cultured cells. The ability of quantitatively assessing functional parameters such as spontaneous beating frequency, depolarization-repolarization patterns and ionic currents magnitude is particularly appealing to drug screening and regenerative medicine applications. Different techniques are available to perform measurements of single cell electrophysiological activity.

To monitor and directly measure extracellular potential of interconnected cellular monolayers *in vitro*, microarrays of electrode (MEAs) have been developed [19]. MEA technology consists of arrays of microelectrodes (made of indium tin oxide or titanium) patterned onto a glass surface that permits simultaneous stimulation and recording of extracellular field potentials generated by cell action potentials. Different electrode patterns (from 8 × 8 up to 16 × 16 grid) can be achieved, allowing for recording signal with high spatial and temporal resolution. Conversely to patch clamp, the most prominent advantage of MEAs relies on the possibility to achieve long-term and noninvasive monitoring of large cell population. For this reason, MEAs have been widely exploited to conduct safety pharmacology studies directly on monolayers of human-induced pluripotent stem cell derived cardiomyocytes, monitoring real-time changes in cardiac action potential duration after drug administration [20]. In order to combine the long-term, noninvasive MEA measurements with the possibility to record intracellular action potentials, protruding gold mushroom-shaped microelectrodes were also developed [21]. This system allows the repeated intracellular recording of cardiomyocyte action potential after an electroporation pulse that temporarily disrupts the cell membrane.

All the above-mentioned technologies are efficient for single cells or interconnected cell monolayer electrical measurements and were successfully integrated in standard 2D culture platforms. However there is an urgent need to integrate electrical measurement systems within advanced microfluidic platforms, where chemical and physical stimulations are tailored to generate *in vitro* complex cardiac models.

## Integrating functional readouts in heart-on-chips

Perspective heart-on-a-chip systems integrate strategies that allow quantitative functional analysis of cardiac tissue parameters. The major advantage of standalone microfluidic devices is that they do not require complicated and potentially disruptive tissue manipulations, while continuously assessing cardiac functional readouts along the whole culture period.

In terms of contractility, muscular thin films (MTF) are 2D bilaminate engineered cultures that recapitulate tissue-scale cardiac contractile behavior. Cardiomyocytes were cultured on deformable thin films that bend in response to the systolic stress generated. Recently, Lind *et al.* [22] upgraded the manufacturing procedure concept and used multimaterial 3D printing technique to fabricate an MTF-inspired heart-on-a-chip. Fully automated acquisition of twitch data was made possible by integrating soft strain gauge sensors within the device. Despite the high automation achieved, MTF strategy does not recapitulate the 3D complex native heart tissue organization.

In microfluidic-based 3D cardiac tissue model configurations, the main data acquisition obstacles are related to highly dense and tightly packed cell configurations that make individual cell analysis challenging. Hence, systems aim at determining the overall mechanical properties of the bulk cardiac tissue. For instance, video-based methods were employed by Mathur *et al.* [23] to analyze time-averaged beating motion and beating kinetics of cardiac microphysiological systems. In general, force quantification of the 3D cardiac constructs by video microscopy is likely to be affected by analysis conditions. Tissue anchors to bulky walls of microdevice that withstand tissue contractions, making moving edges insignificant or undetectable.

In terms of electrical activity recordings, only a few studies report the integration of direct recording systems within heart-on-chip microfluidic platforms. Platinum electroplated planar electrodes in combination with microfluidic networks were used to measure extracellular potentials of single entrapped cardiomyocytes during spontaneous contraction [24]. With a similar approach, patterned Ag/AgCl electrodes placed in two separate microchannels connected by cells are exploited to both stimulate and record cardiac action potential of single-cell or pairs of cardiomyocytes [25].

Microfluidic platforms integrating MEA surfaces have also been developed. Agarose microstructures or PDMS-based culture chambers developed on top of MEAs allow the precise control of spatial cell arrangement, enabling investigations of cardiac electrical activity spatiotemporal development. This technique can be applied to record cardiomyocyte field potential fluctuations in response to drug administration [26], to assess cardiac modulation exerted by sympathetic nervous system [27] or to investigate conduction velocity and cellular physiological functionality after an ischemic event, testing the capabilities of different cell sources to form new gap junctions [28].

Noteworthy, none of the previously described methods (patch clamp or gold mushroom-shaped microelectrodes) allowing for assessing information on intracellular electric potential have yet been coupled to microfluidic platforms. Furthermore, none of the 3D *in vitro* cardiac microfluidic models include electrical activity recording systems.

## Conclusion

The number of organs-on-chip focused on heart physiology is continuously rising and ever more complex microenvironments are tailored for heart cells growth and analysis. The existing literature is widely focused on tailoring specific on-chip environmental cues, particularly electromechanical stimuli, to generate cardiac biological models. However, in order to fulfill the goal of stand-alone platforms for high-throughput analyses of cardiac models performances in basic research or drug screening, a deeper focus in the direction of integrating cardiac cells functional assessments is required. With a successful integration of cardiac output measurement systems, the existing heart-mimicking microfluidic organs-on-chips are expected to pave the way to a new generation of *in vitro* testing capabilities.
